# Supplementary material for: Lifting regenerative barriers promotes epithelial cell fate plasticity supporting lineage conversion
Source: Nat Commun. 2025 Nov 27;16:11305. doi: 10.1038/s41467-025-66446-9 (PMC12722390; doi:10.1038/s41467-025-66446-9)
Supplement: Supplementary file 2 — Reporting Summary [file 41467_2025_66446_MOESM2_ESM.pdf]

nature portfolio

Corresponding author(s): Maria P Alcolea, Maria T. Bejar

Last updated by author(s): Oct 10, 2025

Reporting Summary

Nature Portfolio wishes to improve the reproducibility of the work that we publish. This form provides structure for consistency and transparency in reporting. For further information on Nature Portfolio policies, see our Editorial Policies and the Editorial Policy Checklist.

Statistics

For all statistical analyses, confirm that the following items are present in the figure legend, table legend, main text, or Methods section.

n/a	Confirmed	
☐	☒	The exact sample size (n) for each experimental group/condition, given as a discrete number and unit of measurement
☐	☒	A statement on whether measurements were taken from distinct samples or whether the same sample was measured repeatedly
☐	☒	The statistical test(s) used AND whether they are one- or two-sided<br>*Only common tests should be described solely by name; describe more complex techniques in the Methods section.*
☐	☒	A description of all covariates tested
☐	☒	A description of any assumptions or corrections, such as tests of normality and adjustment for multiple comparisons
☐	☒	A full description of the statistical parameters including central tendency (e.g. means) or other basic estimates (e.g. regression coefficient) AND variation (e.g. standard deviation) or associated estimates of uncertainty (e.g. confidence intervals)
☐	☒	For null hypothesis testing, the test statistic (e.g. F, t, r) with confidence intervals, effect sizes, degrees of freedom and P value noted<br>*Give P values as exact values whenever suitable.*
☒	☐	For Bayesian analysis, information on the choice of priors and Markov chain Monte Carlo settings
☒	☐	For hierarchical and complex designs, identification of the appropriate level for tests and full reporting of outcomes
☐	☒	Estimates of effect sizes (e.g. Cohen's d, Pearson's r), indicating how they were calculated

Our web collection on statistics for biologists contains articles on many of the points above.

Software and code

Policy information about availability of computer code

Data collection	LAS X 4.7.0.28176 and 3.5.5.19976<br>Zen 3.2<br>Arivis 3.5.1
Data analysis	Volocity 6.3, PerkinElmer<br>Volocity 7.0.0 Quorum technologies<br>Zen 3.2<br>Arivis 3.5.1.<br>Microsoft Excel (Microsoft, version 2019)<br>CellRanger (3.1.0)<br>Seurat (3.2.2)<br>g:Profiler (v0.1.8)<br>Harmony (v0.1.0)<br>Monocle 3 (v0.2.3.0)<br>ggplot2 (v3.5.0)<br>GraphPad Prism 10.5.0.<br>cellranger-atac count (v2.2.0)<br>Signac (v1.14.0)

Seurat (v5.3.0)
Cicero (v1.3.9)
Monocle3 (v1.4.26)
EnsDb.Mmusculus.v79_2.99.0
GenomicRanges_1.56.2
ComplexHeatmap version 2.22.0

For manuscripts utilizing custom algorithms or software that are central to the research but not yet described in published literature, software must be made available to editors and reviewers. We strongly encourage code deposition in a community repository (e.g. GitHub). See the Nature Portfolio guidelines for submitting code & software for further information.

Data

Policy information about availability of data

All manuscripts must include a data availability statement. This statement should provide the following information, where applicable:

- Accession codes, unique identifiers, or web links for publicly available datasets
- A description of any restrictions on data availability
- For clinical datasets or third party data, please ensure that the statement adheres to our policy

scRNA and scATAC sequencing data have been deposited in NCBI GEO under accession numbers GSE163218, and GSE303427, respectively. Source data are provided with this paper.

Research involving human participants, their data, or biological material

Policy information about studies with human participants or human data. See also policy information about sex, gender (identity/presentation), and sexual orientation and race, ethnicity and racism.

Reporting on sex and gender	n/a
Reporting on race, ethnicity, or other socially relevant groupings	n/a
Population characteristics	n/a
Recruitment	n/a
Ethics oversight	n/a

Note that full information on the approval of the study protocol must also be provided in the manuscript.

Field-specific reporting

Please select the one below that is the best fit for your research. If you are not sure, read the appropriate sections before making your selection.

[x] Life sciences [ ] Behavioural & social sciences [ ] Ecological, evolutionary & environmental sciences

For a reference copy of the document with all sections, see nature.com/documents/nr-reporting-summary-flat.pdf

Life sciences study design

All studies must disclose on these points even when the disclosure is negative.

Sample size	No statistical methods were used to predetermine sample size. Sample sizes were chosen based on prior experience and are consistent with those commonly reported in the field. A minimum of three independent mice or ex vivo cultures were used in all cases. For image analysis, a minimum of three independent samples were inspected and replicate images were taken per sample for data to be analysed both internally within said sample, and between at least 3 biological replicates.
Data exclusions	Exclusions to data were made only in the case of single cell RNA and ATAC Sequencing analysis to exclude low quality cells as is considered best practice in such analyses. In this case, upper and lower bounds on the distributions of counts, features, mitochondrial and ribosomal RNA were used to remove outlier cells. Cells were included in the analysis if their sequencing depth was over 8750, the number of expressed genes was between 2000 and 8000, and the percentage of mitochondrial and ribosomal DNA was lower than 15% and 45%, respectively Mitochondrial and ribosomal genes were subsequently removed from the matrix. These criteria were established based upon previous preestablished standards combined with analysis from basic QC metrics. Further details are available in the single-cell RNA-seq analysis and single-cell ATAC-seq analysis sections of the manuscript.
Replication	All replicates showed consistent results. All studies were performed with at least 3 mice per group -constituting independent experimental replicates- and several technical replicates for each.
Randomization	Mice were randomly assigned to experimental groups
Blinding	Blinding was not implemented in this study, as all experimental procedures and data analyses were performed using objective, pre-defined

criteria that minimized potential bias.

Reporting for specific materials, systems and methods

We require information from authors about some types of materials, experimental systems and methods used in many studies. Here, indicate whether each material, system or method listed is relevant to your study. If you are not sure if a list item applies to your research, read the appropriate section before selecting a response.

Materials & experimental systems

n/a	Involved in the study
☐	☒ Antibodies
☒	☐ Eukaryotic cell lines
☒	☐ Palaeontology and archaeology
☐	☒ Animals and other organisms
☒	☐ Clinical data
☒	☐ Dual use research of concern
☒	☐ Plants

Methods

n/a	Involved in the study
☒	☐ ChIP-seq
☐	☒ Flow cytometry
☒	☐ MRI-based neuroimaging

Antibodies

Antibodies used

Primary antibodies (well characterised antibodies were used in this study. All are commercially available and validated by the manufacturers)
Caspase-3 active; 1:200; Abcam; ab2302. Validated by manufacturer and previous literature (e.g. Brisson BK, et al. Am J Pathol. 2015)
CD34; 1:100; BD biosciences; 553731. Validation by manufacturer and in Figure 2B (top, skin controls) and previous literature.
CD49f (integrin α6); 1:200; BioLegend; 313610. Validated from literature (e.g. Rosenbluth JM, et al. 2020. Nat Commun.)
HIF1a; 1:100; Novus Biological; NB100-479. Widely validated by supplier and previous literature (e.g. Grubman A, et al. 2021. Nat Commun.)
KRT4; 1:2000; Vector Laboratories; VP-C399. Validated in our previous studies e.g. McGinn J, et al. 2021 Nat Cell Biol.)
KRT10; 1:200; Abcam; ab76318. Validation by manufacturer and in Figure 2B (top, skin controls) and previous literature.
KRT14; 1:1000; BioLegend; 905301. Validation by manufacturer and in Supplementary Figure 1C (top, skin control) and previous literature.
KRT15; 1:1000; Abcam: ab80522. Validation by manufacturer and in Figure 2B (top, skin controls) and previous literature.
KRT17; 1:250; Cell Signalling; 4543S. Validation by manufacturer and in Figure 2B (top, skin controls) and previous literature.
KRT24;1:500; Atlas; HPA022978. Validation by manufacturer and in Figure 2B (top, skin controls) and previous literature.
LHX2; 1:200; Santa Cruz; SC-517243. Validated by manufacturer and previous literature (e.g. Hsu, LC., et al. 2015. Proc Natl Acad Sci)
MMP9; 1:50; R&D Systems; AF909-SP. Validated by manufacturer and previous literature (e.g. Wang D, et al. Int J Mol Sci. 2024)
P63; 1:300; GeneTex; GTX102425 Validated by manufacturer and previous literature (e.g. Neumayer G, et al. Nat Commun. 2024)
PDGFRa; 1:200; R&D Systems; AF1062. Validated by manufacturer (https://www.rndsystems.com/products/mouse-pdgf-ralpha-antibody_af1062)
SCA1; 1:100; Invitrogen; 14-5981-82. Validation by manufacturer and in Figure 2B (top, skin controls) and previous literature..
SCD1; 1:100; R&D Systems; MAB4404. Validation by manufacturer and in Figure 2B (top, skin controls) and previous literature..
SOX2; 1:200; eBioscience; 14-9811-82. Advanced verifications/validation by supplier and widely used in the literature (https://www.thermofisher.com/antibody/product/SOX2-Antibody-clone-Btjce-Monoclonal/14-9811-82)
SOX9; 1:1000; Millipore; AB5535. Well characterised antibody, validated by manufacturer (https://www.merckmillipore.com/GB/en/product/Anti-Sox9-Antibody,MM_NF-AB5535)

Secondary antibodies
Goat IgG 647; Millipore; AP180SA6
Goat IgG 750; Abcam; ab175745
Mouse IgG 647; Invitrogen; A-31571
Mouse IgG 750; Abcam; ab175738
Rabbit IgG 488; Invitrogen; A-21206
Rabbit IgG 555; Invitrogen; A-31572
Rabbit IgG 647; Invitrogen; A-31573
Rabbit IgG 750; Abcam; ab175728
Rabbit IgG; Abcam; ab171870
Rat IgG 647; Abcam; ab150155
Rat IgG 750; Abcam; ab175750

Validation

All antibodies used were validated by the source company and suggested concentrations were used.

Animals and other research organisms

Policy information about studies involving animals; ARRIVE guidelines recommended for reporting animal research, and Sex and Gender in Research

Laboratory animals	Mouse lines used:<br>- mTmG (R26mTmG; stock #007676, Jackson Laboratory)<br>- nTnG (R26nTnG; stock #023537, Jackson Laboratory)<br>- H2B-EGFP (CAG::H2B-EGFP; kindly provided by J. Nichols)<br>- Lgr5-EGFP (Lgr5-EGFP-IRES-creERT2; stock #008875, Jackson Laboratory<br>- Fucci2a (R26Fucci2a, kindly provided by Ian J. Jackson)<br>- R26-CreERT2 (stock #008463, Jackson laboratory)<br>- R26R-Confetti (stock #017492, Jackson laboratory)<br>- Sox9flox/flox (MRC-Harwell)<br>- K14-CreERT mice (stock #005107, Jackson laboratory)<br>- C57BL/6J mice (Charles river strain code, 632)<br>- Nude athymic mice (Charles River strain code, 490)
Wild animals	n/a
Reporting on sex	All experiments comprised a mixture of male and female mice with no disaggregated information for sex collected, as initial preliminary analyses showed no gender-specific differences. For RNA sequencing experiments, only male animals were used in order to avoid confounding effects due to estrous cycle.
Field-collected samples	n/a
Ethics oversight	All experiments were approved by the local ethical review committees of the University of Cambridge and conducted according to Home Office project licenses PPL70/8866 and PP7037913 at the Gurdon Institute and The Anne McLaren Building, University of Cambridge.

Note that full information on the approval of the study protocol must also be provided in the manuscript.

Plants

Seed stocks	n/a
Novel plant genotypes	n/a
Authentication	n/a

Flow Cytometry

Plots

Confirm that:

- [x] The axis labels state the marker and fluorochrome used (e.g. CD4-FITC).
- [x] The axis scales are clearly visible. Include numbers along axes only for bottom left plot of group (a 'group' is an analysis of identical markers).
- [x] All plots are contour plots with outliers or pseudocolor plots.
- [x] A numerical value for number of cells or percentage (with statistics) is provided.

Methodology

Sample preparation	In vitro grown epithelia (oesophageal control, oeOE; and oesophageal-derived skin, oeSKIN) were harvested 3 and 10 days post-culture (D3 and D10, respectively), at which point the epithelia were carefully peeled from their underlying stroma following a 50mM EDTA incubation for 15min at 37°C. A single-cell suspension was obtained by rinsing the peeled epithelia with PBS, and incubating it with 0.5mg/ml Dispase (Sigma) for 5min. EDTA was then added to the samples at a final concentration of 5mM, and the suspension diluted 1/5 by adding FACS Buffer (FB; 2% heat-inactivated Fetal bovine serum (Life Technologies; 26140079), 25mM HEPES (Life Technologies; 15630056)) in order to inhibit Dispase activity. The cell suspension was filtered through a 30µm cell strainer, and centrifuged at 300 g for 10 minutes at 4°C. Cells were finally resuspended in FB containing 1U/µl RNAse Inhibitor.

Instrument	BD FACSAria™ III cell sorter
Software	BD FACSDiva™
Cell population abundance	Samples were sorted for tdTomato fluorescent reporter to select cells from esophageal origin, and EGFP to select cells from skin origin. The only biological contaminants expected were death cells and cell debris. Cell populations can be distinguished from cellular debris using side and forward scatter plotting. Later, dead cells were discarded using DAPI as a live/dead marker and only live cells were used for library preparation and downstream analysis.
Gating strategy	Gating strategy is shown in Supplemental Figure 3.

☒ Tick this box to confirm that a figure exemplifying the gating strategy is provided in the Supplementary Information.
