# Supplementary material for: Lifting regenerative barriers promotes epithelial cell fate plasticity supporting lineage conversion
Source: Nat Commun. 2025 Nov 27;16:11305. doi: 10.1038/s41467-025-66446-9 (PMC12722390; doi:10.1038/s41467-025-66446-9)
Supplement: Supplementary file 1 — Supplementary Information [file 41467_2025_66446_MOESM1_ESM.pdf]

Supplementary Information

Lifting regenerative barriers promotes epithelial cell fate plasticity supporting lineage conversion

Maria T. Bejar[1,2,*], Paula Jimenez-Gomez[1,2], Ilias Moutsopoulos[1,3,#], Harikrishnan Ajith[1,2,#], Bartomeu Colom[4,5], Jamie McGinn[1,5], Seungmin Han[1,6], Greta Skrupskelyte[1,2], Fernando J Calero-Nieto[1,3], Berthold Göttgens[1,3], Irina I. Mohorianu[1], Benjamin D. Simons[1,6,7], Maria P. Alcolea[1,2,*]

Affiliations

[1] Cambridge Stem Cell Institute, Jeffrey Cheah Biomedical Centre, University of Cambridge, Cambridge, UK.

[2] Department of Physiology, Development and Neuroscience, University of Cambridge, Cambridge, UK.

[3] Department of Haematology, Jeffrey Cheah Biomedical Centre, University of Cambridge, Cambridge, UK.

[4] Wellcome Sanger Institute, Hinxton, UK.

[5] Present address: Cambridge Institute of Science, Altos Labs, Cambridge, UK.

[6] Gurdon Institute, University of Cambridge, Cambridge, UK.

[7] Department of Applied Mathematics and Theoretical Physics, Centre for Mathematical Sciences, University of Cambridge, Wilberforce Road, Cambridge, UK.

# These authors contributed equally: Ilias Moutsopoulos, Harikrishnan Ajith

*Corresponding authors: mtb48@cam.ac.uk (M.T.B); mpa28@cam.ac.uk (M.P.A).

Contents:

Supplementary Figures and Legends

- Supplementary Fig.1. 3D heterotypic culture set up and characterisation. Related to Fig. 1.
- Supplementary Fig. 2. *In vitro* validation of oesophageal-to-skin cell fate conversion. Related to Fig. 2.
- Supplementary Fig. 3. *In vitro* oesophageal-to-skin lineage conversion and single-cell RNA sequencing annotation. Related to Figs. 3 and 4.
- Supplementary Fig. 4. Tissue regeneration restricts oesophageal-to-skin lineage conversion. Related to Fig. 4.
- Supplementary Fig. 5. Expression patterns defining major changes in the transcriptional profile along the lineage conversion process. Related to Fig. 5.
- Supplementary Fig. 6. HIF1a-SOX9 modulation. Related to Fig. 6.
- Supplementary Fig. 7. Contribution of oesophageal cells to hair formation *in vivo.* Related to Fig. 7.

Supplementary Figure 1

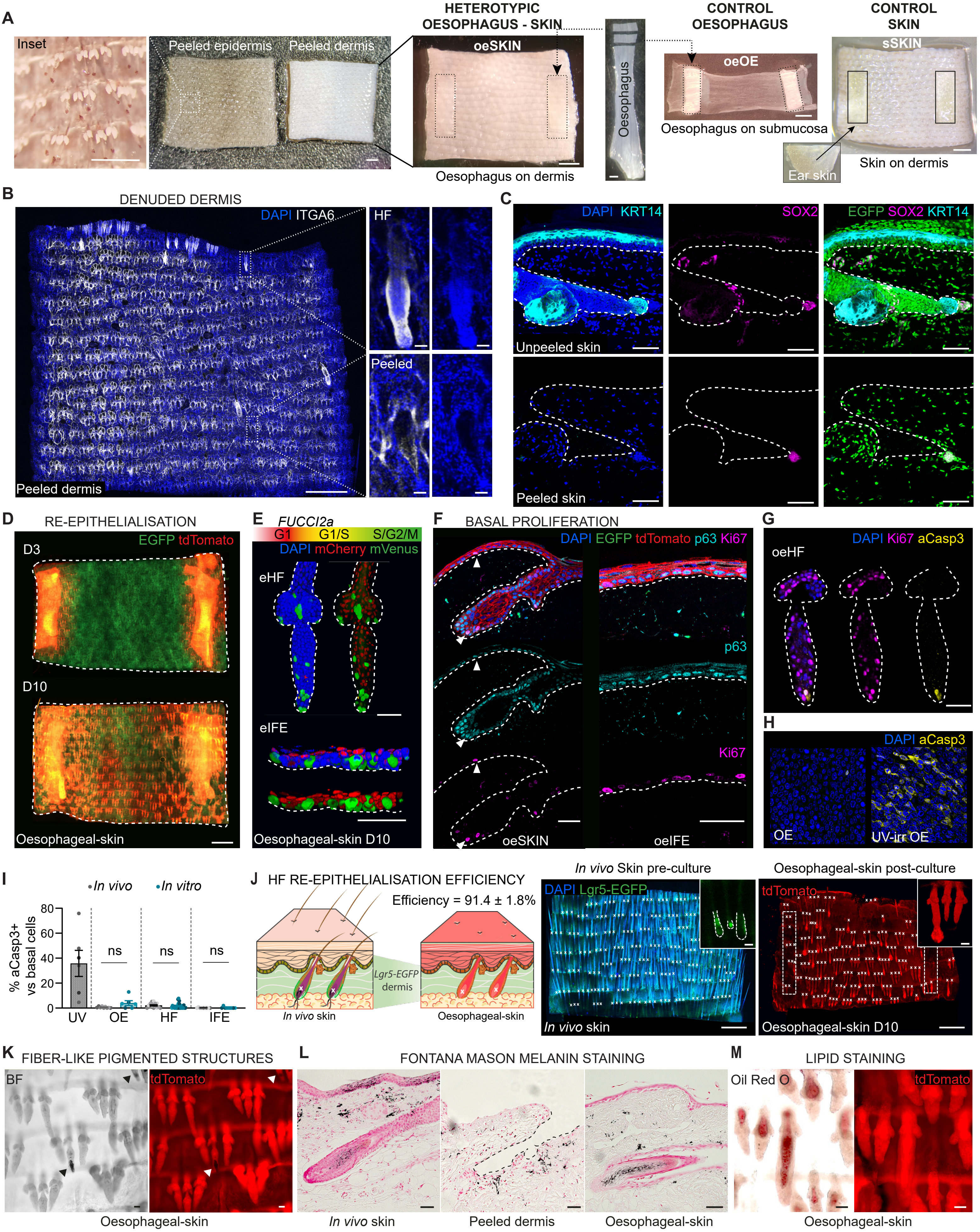

Supplementary Fig.1. 3D heterotypic culture set up and characterisation. Related to Fig. 1.

(A) 3D organotypic culture composite set up. Tail epidermis was peeled away from the underlying stroma (dermis), leaving it denuded of its epithelial compartments, including hair follicles. Inset shows peeled skin epidermis containing hair follicles. Heterotypic cultures were established by placing two strips of oesophageal tissue (black dotted lines) on top of the denuded skin dermis. Controls included oesophageal strips grown on top of denuded oesophageal stroma (referred to as submucosa), and ear skin strips (black solid lines) grown on top of tail dermis. Tissue composites were cultured for up to 10 days. See also Fig. 1B.

(B) Dermis denuded of epidermal compartments. Basal projected view of typical wild-type dermis wholemount following peeling off the epidermis. Insets (dotted lines) show sporadic instances of hair follicles (HFs) remaining in the dermis (right top panels) and a representative socket devoid of HF (right bottom panels). Remaining basement membrane immunostained for integrin α6 (ITGA6) is shown in greyscale.

(C) Tail skin tissue cryosections from *H2B-EGFP* (green) adult mice before (top panels) and after (bottom panels) peeling off the epidermis. The epidermis (positive for KRT14, yellow) was removed in the peeling process, whereas dermal papillae (positive for SOX2, magenta) remained in the HF socket.

(D) Representative basal projected view of typical heterotypic culture wholemounts 3 days (D3, top panel) and 10 days (D10, bottom panel) post-culture as in Figs. 1C-G.

(E) Schematic at the top illustrates the fluorescent expression pattern of cell cycle reporter mouse line *Fucci2a* (mCherry, G1 cells; mVenus, S/G2/M cells). Oesophagi from *Fucci2a* mice were grown for D10 in heterotypic cultures. Side view of oesophageal-derived skin wholemounts, including oesophageal-derived HF (**oe**HF) and oesophageal-derived interfollicular epidermis (**oe**IFE).

(F) Tissue cryosections showing proliferation co-localising with basal marker p63 in oesophageal-derived SKIN (**oe**SKIN) with zoom-in insets showing **oe**IFE. Arrowheads point to examples of proliferating basal cells. P63 in yellow, Ki67 in magenta.

(G) Side view of oesophageal-derived HF wholemounts show low active Caspase 3 expression. Ki67 in magenta, active Caspase3 in yellow.

(H) Top view of *in vivo* oesophageal wholemounts, untreated (OE) or UV irradiated (UV-irr OE) controls, for active Caspase3 analysis. Active Caspase3 in yellow.

(I) Oesophageal-derived skin maintained low levels of Caspase3 activation. Quantification of active Caspase3+ (aCaspase3+) cells in *in vitro* and *in vivo* tissues as a percentage of total number of cells (DAPI+). Ultraviolet (UV)-exposed OE (as in H) was used as a positive immunostaining control. Data presented as mean ± SEM and analysed using a two-tailed unpaired t-test. Points in the graph show individual measurements (grey and blue scale indicate values from each of 3 mice).

(J) Left: Experimental overview. Individual HF units (white crosses) were quantified in donor *Lgr5-EGFP* mouse skin wholemounts (green). Quantified samples were then peeled and dermis used for heterotypic cultures as in Fig. 1B; the resulting oesophageal-derived HF were quantified (white crosses) to calculate the efficiency of oesophageal-derived HF formation. Right: Basal projected views of *in vivo* skin wholemount (left image; inset shows *in vivo Lgr5-EGFP* HFs, delineated by white dashed lines) and oesophageal-derived skin from heterotypic culture at D10 (right image; white dashed lines delineate oesophageal strips). n=3 animals. Created using images provided by Servier Medical Art (https://smart.servier.com), licensed under CC BY 4.0 (https://creativecommons.org/licenses/by/4.0/).

(K) Brightfield (left) and epifluorescent (right) images showing pigment accumulation (indicated by arrows) in oesophageal-derived skin (tdTomato; red).

(L) Fontana-Masson staining showing the presence of melanin in *in vivo* donor skin (left), denuded dermis (middle), and oesophageal-derived skin (right).

(M) Brightfield (left) and epifluorescent (right) images show oil red staining marking the accumulation of lipid droplets in oesophageal-derived hair follicles, denoting the emergence of pilosebaceous-like units.

Fluorescent labels. Blue, DAPI; green, EGFP; red, tdTomato.

Scale bars. 1 mm (A, B, D, and G), 50 µm (insets in B, C, and E).

Mouse lines. Unless otherwise stated, constitutive *H2B-EGFP* and *mTmG* mouse lines were used as sources of skin and oesophageal tissue, respectively.

Supplementary Figure 2

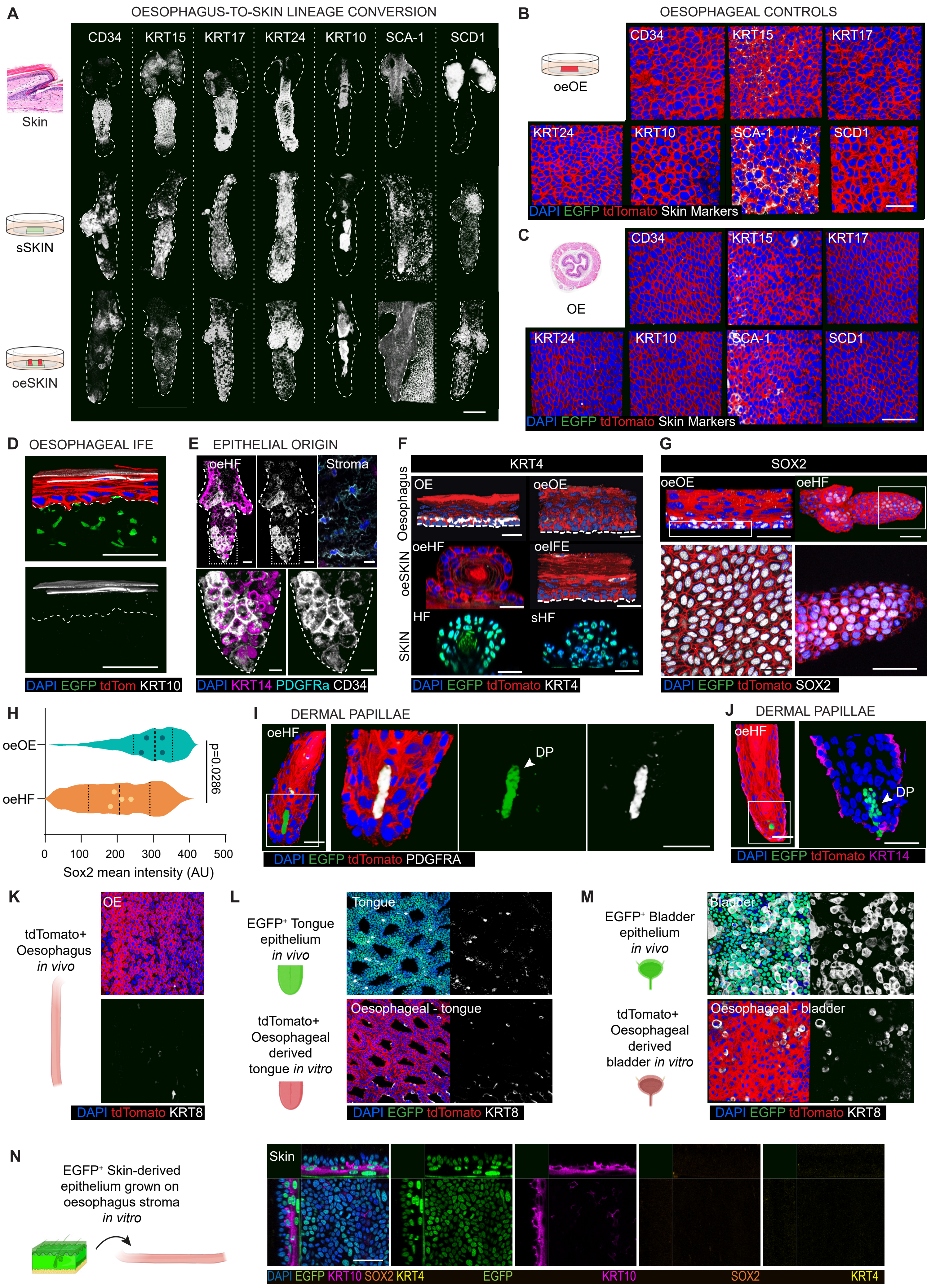

Supplementary Fig. 2. *In vitro* validation of oesophageal-to-skin cell fate conversion. Related to Fig. 2.

(A) Oesophageal-derived skin (**oe**SKIN) shows expression of typical HF markers (greyscale). 3D rendered basal views of wholemount z-stack images showing split greyscale channel from Fig. 2B. Top row shows wholemounts derived from *in vivo* EGFP skin. Middle and bottom rows show heterotypic cultures derived from epidermis (EGFP, **s**SKIN) or oesophagus (tdTomato, **oe**SKIN), respectively.

(B-C) *In vitro* oesophageal controls retain *in vivo* expression profile. Representative 3D rendered basal confocal views of epithelial wholemounts showing typical HF markers in *in vitro* oesophageal epithelium (**oe**OE; B) and *in vivo* oesophagus (OE; C). **oe**OE

(D) Tissue cryosection showing differentiated suprabasal cells in oesophageal-derived interfollicular epidermis (**oe**IFE) express skin marker KRT10. Dermis in green (EGFP).

(E) CD34 expression in **oe**HFs localises to epithelial cells. Single plane confocal images showing oesophageal cells in **oe**HFs co-express KRT14 (basal keratinocyte marker; magenta) and CD34 (hair follicle marker; greyscale). Of note, CD34+ **oe**HF cells were negative for the fibroblast marker PDGFRα (cyan). Oesophageal epithelium was cultured on top of EGFP dermis to discriminate tissue origin.

(F) Oesophageal-derived skin loses expression of typical oesophageal marker KRT4. 3D rendered confocal images showing side views of *in vivo* and *in vitro* oesophagus (OE and **oe**OE), oesophageal-derived skin (**oe**HF and **oe**IFE) and *in vivo* and *in vitro* skin hair follicles (HF and **s**HF, respectively). Labelling shows oesophageal keratinocyte differentiation marker KRT4 in greyscale.

(G-H) Expression of basal oesophageal progenitor marker SOX2 is reduced in oesophageal-derived hair follicles (**oe**HF) compared to oesophageal epithelium *in vitro* (**oe**OE). 3D rendered and single plane inset views showing SOX2 expression in **oe**OE (left panels; F) and **oe**HF (right panels; G). Quantification of SOX2 intensity, as measured by immunofluorescence (a.u.) in **oe**OE and **oe**HFs (H), from (G). The points show individual average values from each biological replicate. Analysis using Mann-Whitney test.

(I-J) EGFP+ dermal papilla cells in oesophageal-derived hair follicles. 3D rendered lateral views of oesophageal-derived hair follicles (**oe**HFs) containing EGFP+ dermal papilla cells positive for PDGFRa staining. DP, dermal papilla.

(K-M) Oesophageal tdTomato+ epithelia (K) was grown on top of denuded tongue and bladder EGFP+ stroma, respectively. The newly oesophageal-derived re-epithelialised tongue (L) and bladder (M) showed both morphological similarities to the original epithelia, and up-regulation of KRT8 expression compared to the oesophageal tissue. (K) depicts oesophageal *in vivo* controls. Top panels in (L) and (M) show *in vivo* controls for each host tissue (bladder and tongue, respectively).

(N) EGFP+ skin epidermis was grown on top of denuded oesophagus. A representative cross-sectional view (xyz plane) of the newly formed epithelium shows no detectable oesophageal markers (SOX2, KRT4) and maintenance of skin-identity marker expression.

Panels A, B, K, L, M and N were created in BioRender. Alcolea, M. (2025) https://BioRender.com/dwxe9n2

Scale bars. 50 µm.

Mouse lines. Unless otherwise stated, constitutive *H2B-EGFP* and *mTmG* mouse lines were used as sources of skin and oesophageal tissue, respectively.

Supplementary Figure 3

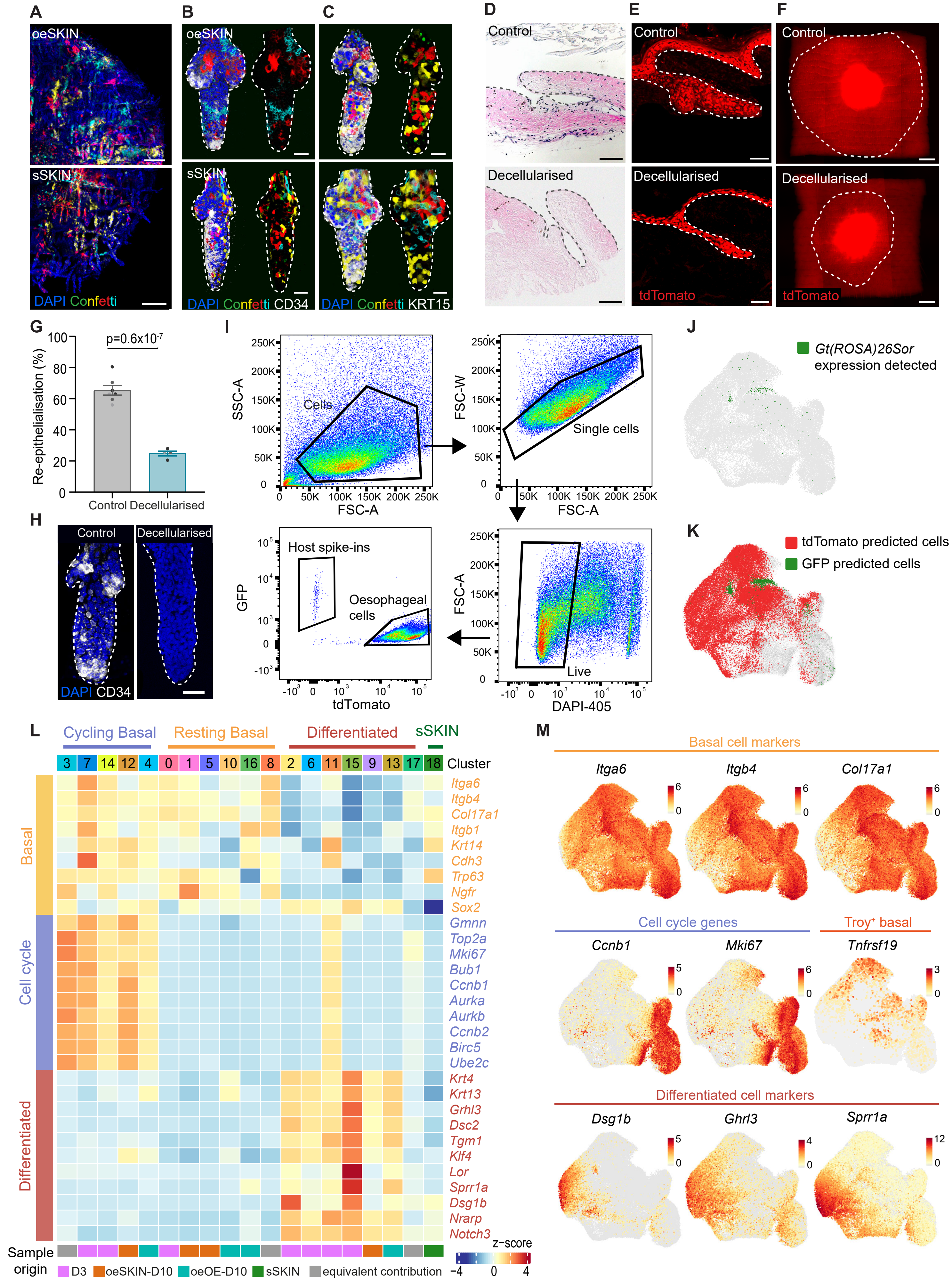

Supplementary Fig. 3. *In vitro* oesophageal-to-skin lineage conversion and single-cell RNA sequencing annotation. Related to Figs. 3 and 4.

(A) 3D rendered tilescans showing epithelial wholemounts of oesophageal origin (**oe**SKIN, top) or skin origin (**s**SKIN, bottom) grown over denuded skin dermis 8 days post-culture. Images show widespread clonal contribution in both oesophageal and epidermal epithelia during the re-epithelialisation process.

(B-C) Lateral 3D rendered views of confocal z-stacks showing polyclonal contribution to KRT15+ and CD34+ populations in both **oe**SKIN and **s**SKIN. KRT15+ and CD34+ cells were not found to be associated to particular clonal events.

(D) H&E sections of control and decellularised dermis showing efficient removal of the cellular component and maintenance of skin appendage structures following decellularisation. Dashed lines indicate basement membrane.

(E) **oe**HF grown on decellularised dermis show impaired morphology. Confocal side view showing **oe**SKIN (tdTomato) cultured on top of denuded alive dermis under control conditions (as per panel B; top) and over decellularised dermis (from panel B; bottom) for 8 days.

(F) Representative basal views of typical heterotypic culture wholemounts (E) showing impaired re-epithelialisation of decellularised dermis compared to controls.

(G) Quantification of re-epithelialisation (tdTomato) in control and decellularised dermis (from D), expressed as percentage of re-epithelialised area relative to total dermis area. Data expressed as mean ± SD and analysed using a two-tailed unpaired t-test.

(H) Decellularised dermis fails to induce CD34 expression in oesophageal-derived HF (**oe**HF). Representative 3D rendered side views showing CD34 expression in **oe**HF grown over control and decellularised dermis (as in C). Out of a total of 82 **oe**HFs, no CD34+ cell was found when exposed to decellularised dermis (n=3 animals). Greyscale, CD34.

(I) Flow cytometry gating strategy for isolation of oesophageal (tdTomato+) and skin (EGFP+) cells from 3D heterotypic cultures. *In vitro* cell suspensions were gated to sort the single viable oesophageal (tdTomato) or skin (EGFP+) populations. Representative FACS plots are shown.

(J) Cluster 18 contains **s**SKIN reference control cells (EGFP+). UMAP showing enriched *Gt(ROSA)26Sor* gene expression in cluster 18. Please note *Gt(ROSA)26Sor* gene is disrupted in oesophageal cells due to insertion of the *nTnG* cassette.

(K) UMAP showing spatial distribution of the predicted tdTomato+ and EGFP+ cells. Prediction is based on a semi-supervised model classifier that extends transcriptomic signatures of the cells with detected tdTomato and EGFP transcripts to unlabelled cells (tdTomato/EGFP transcripts not detected). Grey indicates unassigned cells.

(L) Heatmap showing expression of representative marker genes for basal cells, cell cycle, and differentiation for the 19 clusters shown in Fig. 4B. Expression levels were log-transformed normalised followed by averaging across cells in the same clusters and scaling.

(M) UMAPs showing expression of representative markers for basal, cell cycle and differentiated cells from (L). Colour bars of UMAPs indicate log2- transformed normalised expression levels.

Scale bars. 50 μm.

Supplementary Figure 4

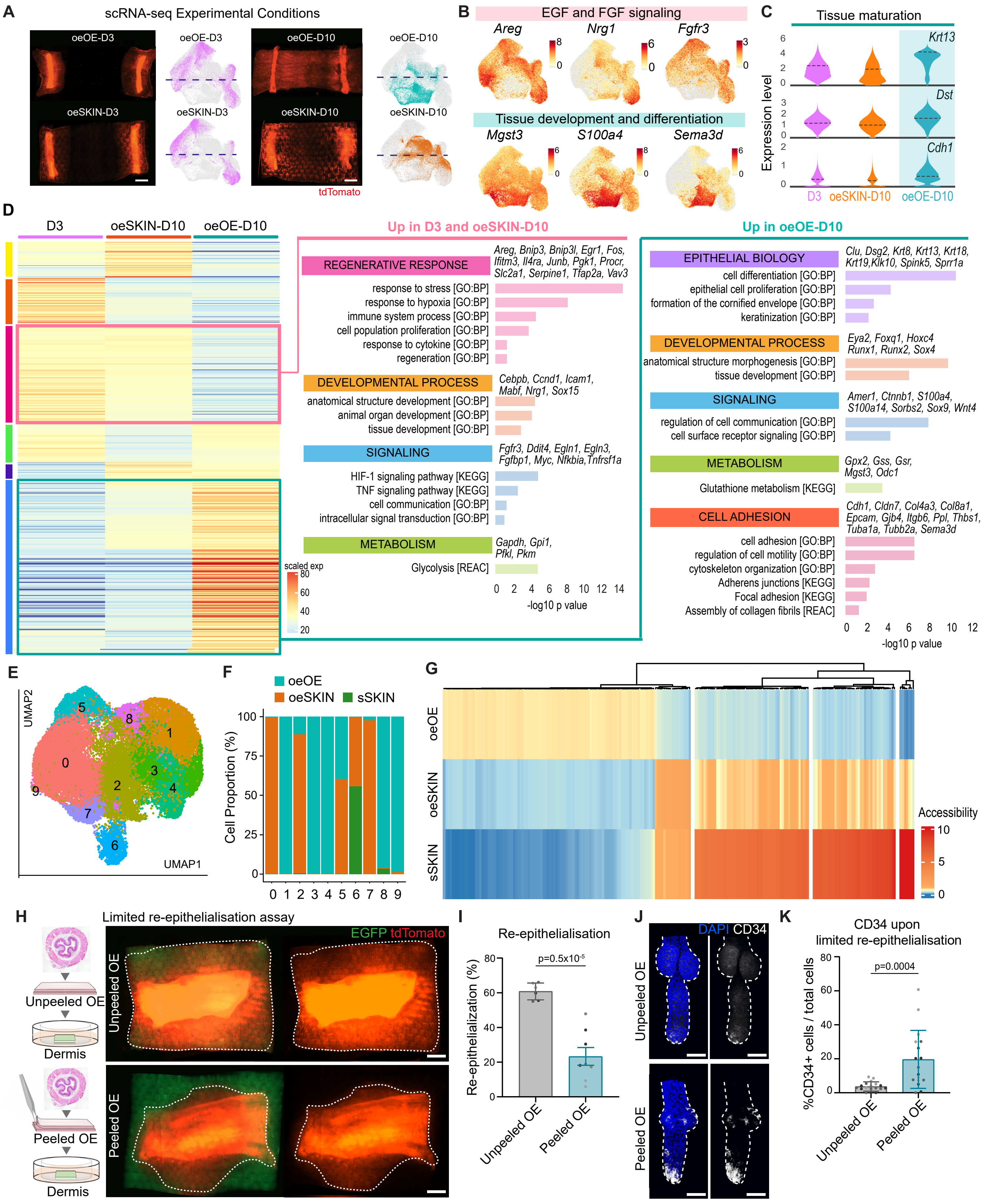

Supplementary Fig. 4. Tissue regeneration restricts oesophageal-to-skin lineage conversion. Related to Fig. 4.

(A) Representative images of **oe**OE and **oe**SKIN heterotypic cultures at D3 and D10 post-culture are shown adjacent to their respective UMAPs cell distribution. Three distinctive transcriptional patterns are revealed. **oe**OE/**oe**SKIN-D3, **oe**SKIN-D10 and **oe**OE-D10. tdTomato (red), epithelium of oesophageal origin.

(B) UMAPs showing expression of selected genes related to key biological processes from transcriptional pattern analysis (see also Figure 4). DEGs upregulated in **D3+oeSKIN-D10** (pink); DEGs upregulated in **oeOE-D10** (cyan). Colour bars of UMAPs indicate log2-transformed normalised expression levels.

(C) Violin plots showing the expression distribution of genes associated with tissue maturation in basal cells across the three transcriptional patterns (genes enriched in **oeOE-D10** shaded cyan). Data is presented as log2-transformed normalised expression levels. Dashed lines represent mean values.

(D) Heatmap showing the expression of individual DEGs in basal cells across patterns (as per Figs. 4H,I). Log2 transformed normalised expression levels were averaged and scaled across all cells belonging to each group. Scale bar denotes expression range. The tables on the right show the transcriptional signature of the two major transcriptional patterns identified (pink outline denotes genes upregulated in **D3+oeSKIN-D10**; cyan outline comprises genes upregulated in **oeOE-D10**). Tables contain a selected compilation of representative DEGs in relevant Gene Ontology Biological Process (GO:BP), Kyoto Encyclopedia of Genes and Genomes (KEGG) and Reactome (REAC) databases, with their corresponding p-values (colour bars). Closely related terms are grouped together. See Source Data for complete gene enrichment analysis results.

(E) UMAP representing cell clusters based on louvain clustering (10 clusters in total labelled 0 to 9) from single-cell ATAC seq data (as in Fig.5A).

(F) Bar chart showing the percentage of cells from each sample within each cluster of the scATAC-seq UMAP (as in E).

(G) Heatmap plotting normalised accessibility for the top 100 differentially accessible loci among samples shows changes in **oe**SKIN chromatin accessibility compared to **oe**OE.

(H) Left: schematic depicting the strategy for the limited re-epithelialisation assay. Controls were performed with unpeeled oesophageal tissue (top; as in Fig. 1B), while limited assay used peeled oesophageal epithelium (lacking stroma) to re-epithelialise the denuded dermis (bottom). Right: Representative tilescan confocal images of controls (top) and limited re-epithelialisation assays (bottom) following 8 days in culture. tdTomato (red) denotes oesophageal origin; EGFP (green) denotes skin origin. Dotted lines delineate oesophageal growth at day 8. Some elements created with images provided by Servier Medical Art (https://smart.servier.com), licensed under CC BY 4.0 (https://creativecommons.org/licenses/by/4.0/).

(I) Quantification of oesophageal re-epithelialisation from (H), expressed as percentage of area re-epithelialised by oesophageal cells (tdTomato, red) relative to the dermis area. A minimum of 6 cultures were quantified from out of 3 animals. Data expressed as mean ± SD and analysed using a two-tailed unpaired t-test. The points show individual measurements, greyscale indicates values from each of 3 mice.

(J) 3D confocal side views of oesophageal-derived skin wholemounts showing typical CD34 expression in **oe**HFs derived from either unpeeled oesophagus (controls) or peeled oesophageal epithelium. Dashed lines delimit hair follicles borders.

(K) Quantification of CD34+ cells per **oe**HF relative to the total number of DAPI+ cells (from J). A minimum of 1700 cells were quantified out of 17 **oe**HF and 3 mice. The points show individual measurements, greyscale indicates values from each of 3 mice. Data expressed as mean ± SD and analysed using a two-tailed unpaired t-test.

Fluorescent labels. Blue, DAPI; green, EGFP; red, tdTomato; Greyscale CD34.

Scale bars. 1mm (A, H), 50 µm (J)

Mouse lines. Unless otherwise stated, constitutive *H2B-EGFP* was used as sources of skin and *nTnG* (A, B) and *mTmG* (C-G) mouse lines were used as sources oesophageal tissue.

Supplementary Figure 5

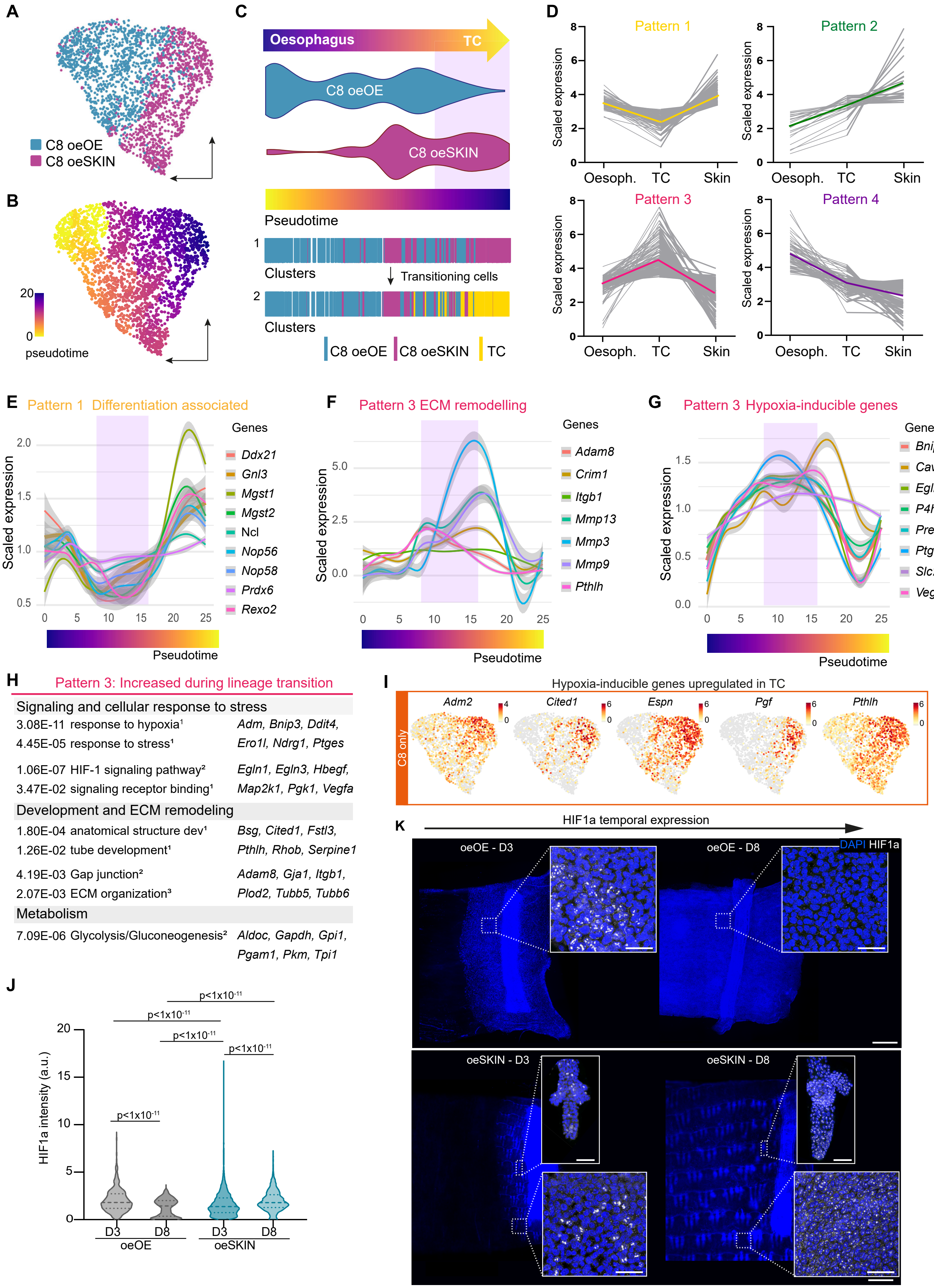

Supplementary Fig. 5. Expression patterns defining major changes in the transcriptional profile along the lineage conversion process. Related to Fig. 5.

(A) UMAP representation of C8 cells from **oe**OE and **oe**SKIN after re-clustering. Colours show the sample origin.

(B) Pseudotime trajectory for C8 cells only on the UMAP representation. Colour bar denotes pseudotime axis.

(C) Violin plots showing the distribution of cells along the pseudotime trajectory reveal a divergent profile between control cells (**oe**OE) and oesophageal-derived skin cells (**oe**SKIN). Expression-driven rug plots illustrate the position of individual cells along the pseudotime trajectory. Cells highlighted in yellow correspond to transitioning cells (TC, as in Fig. 5F), confirming that the divergent **oe**SKIN cells represent those at the transition between oesophageal and skin identity.

(D) Distinctive patterns of gene expression in basal cells across different pseudotime states. Grey, relative expression profile of individual genes belonging to each pattern. Solid coloured lines, for each pattern, averaged values of each pseudotime state (in Fig. 5G). To calculate the relative expression profiles, log-transformed normalised expression levels were averaged and scaled across all cells at each pseudotime state.

(E-G) Expression of representative genes (from Fig. 5G) along the pseudotime trajectory. Pattern 1, showing genes with reduced expression in transitioning cells (E); pattern 3, depicting representative extracellular matrix remodelling (F), and hypoxia-inducible (G) genes. Data is presented as auto-scaled, log2-transformed normalised expression levels smoothed using a generalised additive model. The bar at the bottom denotes pseudotime axis (as per Fig. 5F).

(H) Table summarising some of the most significant terms and genes enriched in Pattern 3 denoting genes enriched in transitioning cells. [1]Gene Ontology Biological Process (GO:BP), [2]Kyoto Encyclopedia of Genes and Genomes (KEGG) and [3]Reactome (REAC). p-values are shown.

(I) C8 re-clustering UMAP projections showing expression of hypoxia-inducible genes (Pattern 3, as in Fig. 5K) up-regulated in transitioning cells (TC). Colour bars of UMAPs indicate log2-transformed normalised expression levels.

(J) Quantification of HIF1a intensity, as measured by immunofluorescence (a.u), in oesophageal epithelium (**oe**OE) and oesophageal skin (**oe**SKIN) grown *in vitro* for 3 and 8 days (D3 and D8). Data was analysed using two-way ANOVA followed by Tukey's multiple comparisons test. Not significant p-values not shown (p-value>0.05), n=3.

(K) Tilescan confocal images of **oe**OE and **oe**SKIN at day 3 (D3) and day 8 (D8) showing HIF1a expression. Insets show zoomed in images denoting the persistent HIF1a expression of **oe**SKIN samples 8 days post-culture (D8).

Fluorescent labels. Blue, DAPI; Greyscale, HIF1a (K).

Scale bars. 1mm (K) and 50 µm (K insets).

Mouse lines. Constitutive *H2B-EGFP* mice were used as source of tail skin and C57BL/6 wild-type were used as source of oesophageal tissue.

Supplementary Figure 6

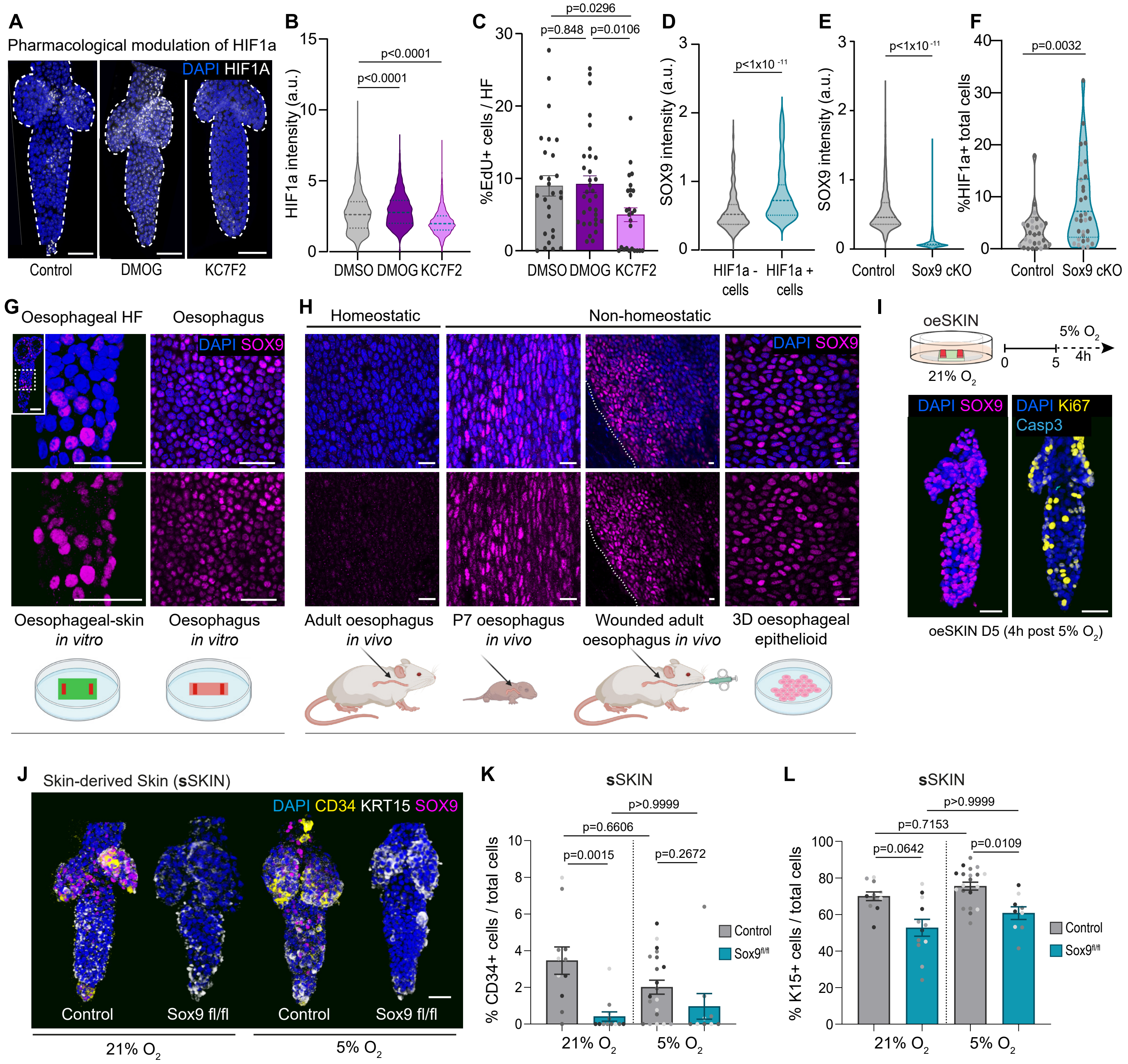

Supplementary Fig. 6. HIF1a-SOX9 modulation. Related to Fig. 6.

(A) Representative 3D confocal side views of **oe**SKIN wholemounts showing HIF1a expression in oesophageal-derived HF (**oe**HF) following treatment with HIF1a activator (DMOG; middle) or HIF1a inhibitor (KC7F2; right) for 8 days. Vehicle control were DMSO treated.

(B) Quantification of HIF1a intensity, as measured by immunofluorescence (a.u.), in individual cells from **oe**HF following HIF1a pharmacological treatment (from A). A minimum of 2,000 cells were quantified out of 18 **oe**HF and 3 animals.

(C) Percentage of EdU+ cells per **oe**HF following treatment with HIF1a activator (DMOG) or HIF1a inhibitor (KC7F2) for 8 days. Vehicle controls were DMSO treated. Data was analysed using Kruskal-Wallis followed by multiple comparison test. n=4

(D) Violin plot showing SOX9 intensity, as measured by immunofluorescence (a.u.), in individual HIF1a positive and negative cells in **oe**HF. A total of 290 HIF1a+ cells were quantified out of 27 **oe**HF (~8,000 DAPI+ cells) and 3 animals.

(E) SOX9 intensity quantification, as measured by immunofluorescence (a.u.), in individual **oe**HF cells derived from control and *Sox9* cKO mice (as per Figs. 6C). A minimum of 8,000 cells were quantified out of 27 **oe**HF and 3 animals.

(F) Genetic inactivation of *Sox9* (*Krt14*^{CreERT}/ *Sox9*$^{fl/fl}$ mice) promotes HIF1a expression in oesophageal-derived HFs (**oe**HF). The percentage of HIF1a+ cells per **oe**HF, measured by immunofluorescence (a.u.), is shown relative to total cells (DAPI+). A minimum of 8,000 cells were quantified out of 27 **oe**HF and 3 animals. The points show individual measurements (greyscale indicates values from each of 3 mice).

(G-H) SOX9 expression pattern in oesophageal tissue. 3D rendered confocal images showing SOX9 expression in basal cells in oesophageal epithelium under different experimental conditions. (G) Heterotypic culture system (as illustrated in Figure 1B); *in vitro* oesophageal-derived HF (**oe**HF) (left) and in vitro oesophageal epithelium (right). (H) Additional conditions included *in vivo* oesophageal tissue denoting adult homeostasis (left), as well as other non-homeostatic conditions; in vivo neonatal tissue (harvested at postnatal day 7, P7), adult tissue following micro-endoscopic biopsy wounding *in vivo*, and 3D Epithelioid culture. Diagrams at the bottom depict the different experimental conditions analysed for SOX9 expression.

(I) Representative 3D rendered side views of oesophageal-derived hair follicles (**oe**HFs) show increased SOX9 expression upon exposure to low oxygen concentration (5% O_2, 4 hours). Note, cells remain viable (Ki67+), with no active Caspase3 positive cells detected.

(J) Control **s**SKIN samples from Figure 6G. Induced *Krt14*^{CreERT}/ *Sox9*$^{fl/fl}$ mice were induced to conditionally delete *Sox9* expression, and **s**SKIN heterotypic cultures were established. Cultures at day 5 post-culture were exposed to 5% or 21% oxygen for 4h, and then returned to normal culture conditions (21% O_2) until tissue collection at day 8. 3D confocal side views of tissue wholemounts showing CD34, KRT15 and SOX9 expression in **s**HF.

(K,L) Quantification (from J) of CD34+ and KRT15+ cells per **s**HF, expressed as a percentage of the total number of cells (DAPI+). A range of 3,200-6,600 cells were quantified out of 12-28 **s**HF and 4 mice. The points show individual measurements (greyscale indicates values from each of the mice). Data expressed as mean ± SEM. Analysis using Kruskal-Wallis followed by Dunn's multiple comparisons test.

Violin plots show median (dashed line) and quartiles (dotted lines). Data from (B-C) was analysed using Kruskal-Wallis ANOVA test followed my multiple comparisons, while data from (D-F) was analysed using two-tailed unpaired Mann-Whitney test.

Panels G and H were created in BioRender. Alcolea, M. (2025) https://BioRender.com/fu23gnv

Fluorescent labels. Blue, DAPI; Greyscale, HIF1a; magenta, SOX9.

Scale bars. 50 µm.

Mouse lines. Constitutive *H2B-EGFP* mice were used as source of tail skin. C57BL/6 wild-type (A-D) and *K14*^{CreERT}/ *Sox9*$^{fl/fl}$ mice (E, F, J-L) were used as source of oesophageal tissue.

Dashed lines delimit hair follicles borders.

Supplementary Figure 7

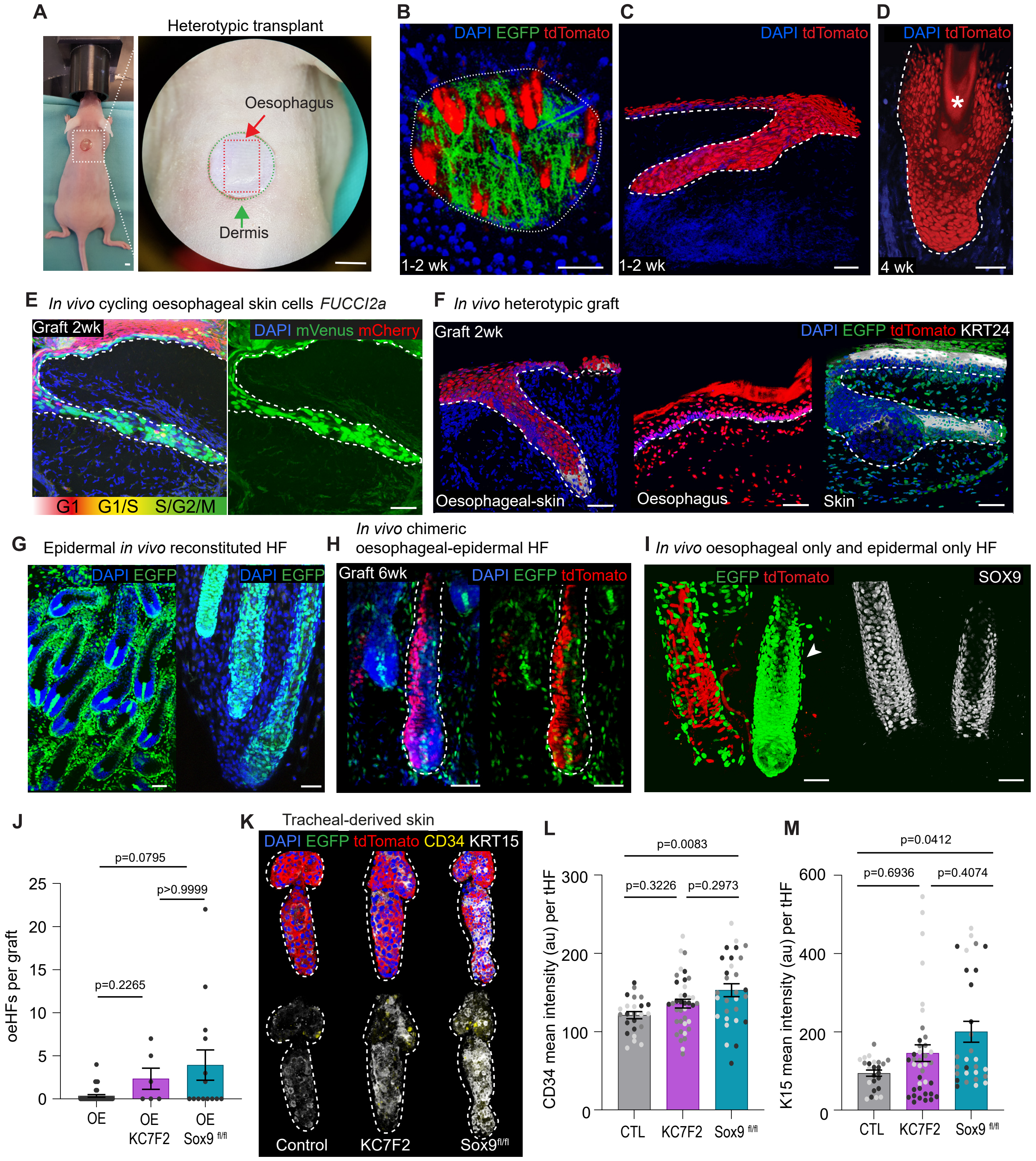

Supplementary Fig. 7. Contribution of oesophageal cells to hair formation *in vivo.* Related to Fig. 7.

(A) Oesophageal-dermal heterotypic tissue implants were prepared overlaying a 4x8mm oesophageal tissue strip from tdTomato expressing mice on top of an 8mm diameter piece of peeled dermis from the tail skin of EGFP expressing mice. The implants were grafted onto the back skin of female nude recipients after an 8mm diameter full-thickness excision punch (inset; right). Contribution of oesophageal cells (tdTomato) to skin/hair follicle regeneration was analysed 1 to 4 weeks after transplant.

(B) Representative 3D rendered basal view of a full thickness skin wholemount 1-2 weeks post-graft (as in A), showing the *in vivo* formation of oesophageal-derived skin (tdTomato). Grafted dermis obtained from *H2B-EGFP* mouse tail skin.

(C-D) Side views showing oesophageal-derived HF structures from *in vivo* heterotypic skin grafts. Full thickness skin graft (tissue cryosections; C), 3D rendered confocal inset (D). Asterisk in (D) denotes structure reminiscent of a hair shaft.

(E) Representative confocal side view showing cycling oesophageal-derived skin cells *in vivo* 2 weeks after heterotypic skin graft transplant (tissue cryosections). Schematic at the bottom illustrates the fluorescent expression pattern of cell cycle reporter mouse line *Fucci2a* (mCherry, G1 cells; mVenus, S/G2/M cells). Blue, DAPI; red, mCherry; green, mVenus. n=2 animals.

(F) Confocal side views showing typical oesophageal-derived HF structures from *in vivo* heterotypic skin graft 2 weeks after transplant, compared to oesophageal and skin controls. Full thickness skin graft/tissue cryosections reveal upregulation of the HF marker KRT24 in oesophageal-derived HFs (red, tdTomato) *in vivo*. EGFP (green) denotes skin origin.

(G-H) 3D rendered confocal side views of full thickness skin wholemounts 6 weeks after cell grafting (as in Figure 7A), showing EGFP epidermal-derived hair follicles (G) and oesophageal-skin chimeric hair follicles (H). tdTomato (red) denotes oesophageal origin); EGFP (green) denotes skin origin.

(I) Representative confocal side views showing localisation of SOX9 expression in epidermal control (green, EGFP) and oesophageal-derived (red, tdTomato) hair follicles in 6 weeks skin graft *in vivo*.

(J) Quantification of the number of oesophageal-derived hair follicles found per graft as in Fig. 7F. Data was analysed using Kruskal-Wallis ANOVA test followed by Dunn's multiple comparisons. (OE n=34; KC7F2-treated OE n=6; $Sox9^{fl/fl}$ OE n=12).

(K) Tracheal-epithelium (tdTomato+) was used to set up heterotypic cultures on top of denuded dermis (EGFP+) as described for the oesophagus in Fig.1B. 3D rendered basal views of wholemount z-stack images showing tracheal-derived HFs up-regulating the expression of HF markers CD34 and KRT15.

(L-M) Quantification (from K) of CD34+ and KRT15+ mean intensity per tracheal-derived HF shows an increased acquisition of HF marker expression in tracheal-derived HFs from induced $Krt14^{CreERT}/Sox9^{fl/fl}$ mice compared to controls. Data expressed as mean ± SEM. Analysis using Kruskal-Wallis followed by Dunn's multiple comparisons test (24 to 38 tracheal-derived hair follicles were analysed from a total of 3 mice).

Dotted line indicates graft-host skin interface. Dashed lines delimit hair follicles borders.

Fluorescent labels. Blue, DAPI; green, EGFP; red, tdTomato; greyscale, KRT24 (F), SOX9 (I), KRT15 (K); yellow, CD34 (K).

Scale bars. 4mm (A), 1mm (B), and 50µm (C-I).

Mouse lines. Constitutive *H2B-EGFP* (A, B, G-I) or C57BL/6 wild-type mice (C-F) were used as source of skin. *mTmG* (B), *Fucci2a* (E) and *nTnG* (C-D, F, H-I) were used as source of oesophageal tissue. *mTmG* and *mTmG* / $Krt14^{CreERT}/Sox9^{fl/fl}$ mice were used as source of tracheal tissue. Nude mice were used as graft recipients.
